# Preclinical and Clinical Observations Implying Combination Therapy to Enhance the Efficacy of the Her-2/neu B-Cell Peptide-Based Vaccine HER-Vaxx and to Prevent Immune Evasion

**DOI:** 10.3390/ijms25010287

**Published:** 2023-12-24

**Authors:** Joshua Tobias, Sandra Högler, Martin Raigel, Diego Shih-Chieh Lin, Yee Chao, Lukas Kenner, Erika Garner-Spitzer, Sharon Yavrom, Nicholas J. Ede, Christoph C. Zielinski, Michael Kundi, Ursula Wiedermann

**Affiliations:** 1 Institute of Specific Prophylaxis and Tropical Medicine, Center for Pathophysiology, Infectiology and Immunology, Medical University of Vienna, 1090 Vienna, Austria; erika.garner-spitzer@meduniwien.ac.at; 2 Institute of Pathology, Unit of Laboratory Animal Pathology, University of Veterinary Medicine Vienna, 1210 Vienna, Austria; sandra.hoegler@vetmeduni.ac.at (S.H.);; 3 Department of Oncology, Taipei Veterans General Hospital, Taipei 11217, Taiwan; diegolin@vghtpe.gov.tw (D.S.-C.L.); ychao@vghtpe.gov.tw (Y.C.); 4 Department of Experimental Pathology, Medical University of Vienna, 1090 Vienna, Austria; lukas.kenner@meduniwien.ac.at; 5 Imugene Limited, Sydney, NSW 2000, Australia; syavrom@imugene.com (S.Y.); nede@imugene.com (N.J.E.); 6 Central European Cancer Center, Wiener Privatklinik, and Central European Cooperative Oncology Group (CECOG), 1090 Vienna, Austria; christoph.zielinski@cancer-center.cc; 7 Department of Environmental Health, Center for Public Health, Medical University of Vienna, 1090 Vienna, Austria; michael.kundi@meduniwien.ac.at

**Keywords:** PD-L1, Her-2/neu, expression, preclinical and clinical settings, immune evasion, combination therapy

## Abstract

Her-2/neu-targeting therapy by passive application with trastuzumab is associated with acquired resistance and subsequent metastasis development, which is attributed to the upregulation of tumoral PD-L1 expression and the downregulation of Her-2/neu. We aimed to investigate this association, following active immunization with our recently constructed B-cell peptide-based Her-2/neu vaccines in both preclinical and clinical settings. Immunohistochemistry (IHC), fluorescence in situ hybridization (FISH), and combined positive score (CPS) were applied to evaluate Her-2/neu and PD-L1 expression using a murine syngeneic tumor model for Her-2/neu lung metastases and tumor biopsies from a gastric cancer patient with disease progression. A significant and concomitant reduction in Her-2/neu and the upregulation of PD-L1 expression was observed in vaccinated mice after 45 days, but not after 30 days, of metastases development. A significant increase in tumor-infiltrating B lymphocytes was observed at both time points. The downregulation of Her-2/neu and the upregulation of PD-L1 were observed in a patient’s primary tumor at the disease progression time point but not prior to vaccination (Her-2/neu IHC: 3 to 0, FISH: 4.98 to 1.63; PD-L1 CPS: 0% to 5%). Our results further underline the need for combination therapy by targeting PD-L1 to prevent metastasis formation and immune evasion of Her-2/neu-positive and PD-L1-negative tumor cells.

## 1. Introduction

Her-2/neu, a member of the epidermal growth factor receptor (EGFR) family, is an attractive tumor-associated target antigen for cancer therapy because of its overexpression and association with aggressive biological cancer phenotypes, reduced survival, poor response to traditional chemotherapy, and, consequently, unfavorable prognosis [1]. The use of trastuzumab (Herceptin^®^), a monoclonal antibody (mAb) blocking Her-2/neu downstream signaling and inducing antibody-dependent cellular cytotoxicity (ADCC), has significantly improved the survival rate of patients with Her-2/neu-positive carcinomas [2].

Based on the results of the phase III ToGA (trastuzumab for Gastric Cancer) trial [3], cisplatin/fluoropyrimidine plus trastuzumab, with the latter binding to extracellular domain IV of Her-2/neu, blocking Her-2/neu signaling, and mediating ADCC, has become a standard first-line treatment for patients with Her-2/neu-positive advanced gastric cancers. However, resistance to trastuzumab restricts its therapeutic efficacy in patients with both breast and gastric cancers [4]. Programmed cell death ligand-1 (PD-L1) is an immune checkpoint (IC) expressed on various tumor cells [5], and its binding to its receptor Programmed cell death 1 (PD-1), which is expressed in various immune cells, including tumor-infiltrating T cells [6], results in immune evasion [4]. The upregulation of PD-L1 in Her-2/neu-expressing tumors is considered a mechanism of acquired resistance to trastuzumab [4].

The success of passive immunotherapy with trastuzumab has paved the way for the identification of B-cell peptides/binding epitopes of mAbs for use in active immunization [7]. Using a computer algorithm, we identified and used three single peptides (P4, P6, and P7) and a formulated Her2 vaccine (HER-Vaxx), comprising B-cell peptides from the trastuzumab binding site, conjugated to the cross-reacting material 197 (CRM197) as a carrier protein and used them together with the adjuvant Montanide [8]. By employing an Her-2/neu lung metastasis mouse model [9], the antitumor effect of the vaccine, alone or together with the mimotope of pertuzumab as a multi-peptide vaccine, was recently shown [9]. In a phase Ib dose-escalation study (NCT02795988), HER-Vaxx was shown to be safe and prolong progression-free survival in patients with Her2-overexpressing gastro-esophageal junction (GEJ) and gastric cancer (GC) [10]. Based on the success of this evaluation, the highest dose (50 µg) of the vaccine was selected [10] and evaluated in a phase II randomized controlled trial to compare the effects of the vaccine plus chemotherapy versus chemotherapy alone in patients with GC and GEJ adenocarcinoma [11]. The vaccination has shown a robust anti-HER-2/neu IgG antibody response, significantly correlating with the reduction of tumor sizes, and a statistically significant overall survival benefit in vaccinated patients compared to patients treated with chemotherapy alone [11,12,13].

Considering the fact that pertuzumab, an additional therapeutic mAb against Her-2/neu, is used simultaneously together with trastuzumab for the treatment of patients with metastatic Her-2/neu-overexpressing breast cancer, we formulated a multi B-cell peptide vaccine comprising HER-Vaxx and the mimotope/binding epitope of pertuzumab. This vaccine showed significant prevention of Her-2/neu-expressing lung metastasis formation in a mouse model, in association with the upregulation of PD-L1 and the downregulation of Her-2/neu expression in the tumors of the treated mice [9,14].

Here, we aimed to further investigate the association observed in the preclinical setting. Additionally, we took this aim one step further to examine the occurrence of such observations in a clinical setting by examining the primary tumor of a patient from the phase Ib trial of HER-Vaxx (NCT02795988) who had disease progression after prolonged treatment with HER-Vaxx. The comparison of Her-2/neu-targeted vaccination in both clinical and preclinical settings further sheds light on potential immune evasion after targeting the receptor, further emphasizing the effective tumor elimination and prevention of tumor immune evasion using combination therapy.

## 2. Results

### 2.1. Her-2/neu-Targeted Therapy Upregulates PD-L1 Expression in Association with Loss of Her-2/meu Expression—Preclinical Observation

We formulated a multi-peptide vaccine for targeting the binding sites of trastuzumab and pertuzumab using HER-Vaxx and a B-cell epitope (mimotope; JTMP), respectively [8]. Applying a mouse model of Her-2/neu lung metastasis, we previously demonstrated, that 4 weeks after vaccination with a multi-peptide vaccine, a significant decrease in the size of metastasized lungs was observed [9]. We further showed that the antitumor effect was associated with the upregulation of PD-L1 and the downregulation of Her-2/neu expression, reflected by a significant ratio of PD-L1-positive to Her-2/neu-positive tumors in the lungs [9].

**Figure 1:**
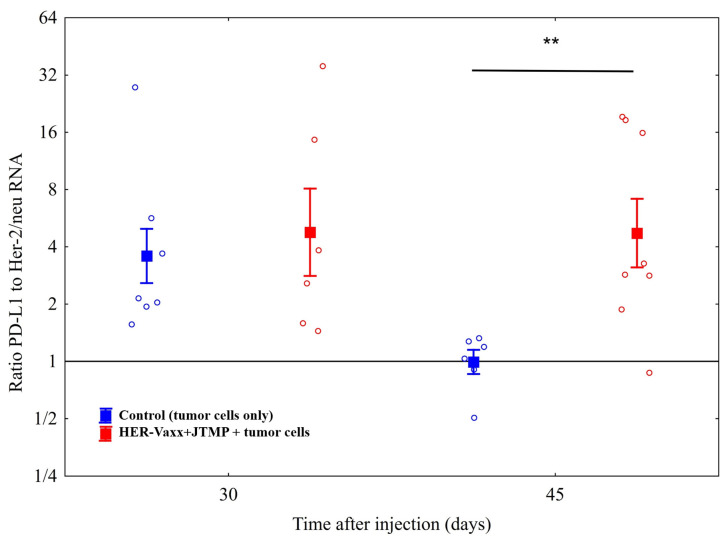
The ratio of PD-L1 to Her-2/neu RNA in metastasized lungs of mice. Mice (*n* = 8/group), either administered with PBS (controls) by injection or actively immunized three times with the multi-peptide vaccine (HER-Vaxx plus the mimotope (JTMP) of pertuzumab), followed by tail-vein injection with tumor cells expressing human Her-2/neu [8,9] and sacrificed on days 30 and 45 following the tumor cell injection. Means and 95% confidence intervals are shown and estimated using a generalized linear model with all the time points and both mRNA levels evaluated simultaneously. The ratio of PD-L1 to Her-2/neu RNA from vaccinated and control (tumor cells only) mice were calculated based on the RT-PCR Ct values relative to β-actin, using the formula 2^−∆Ct^. Comparisons were with linear contrasts (Sidak-corrected). (**, *p* < 0.01).

In this study, we aimed to further investigate the observed upregulation of PD-L1 associated with the loss of Her-2/neu expression at the cellular level. Mice were either only tail-vein injected with 5 × 10^4^ of BALB/c-derived mammary carcinoma cells expressing human Her-2/neu, that is, control mice, or were actively immunized with the multi-peptide vaccine (HER-Vaxx plus JTMP, 50 µg/dose) [9] prior to tail-vein injection of the tumor cells. To examine the kinetics of the previously observed upregulation of PD-L1 and the downregulation of Her-2/neu expression, the control and treated mice were sacrificed 30 and 45 days after the injection and their lungs were excised for evaluation of PD-L1 and Her-2/neu expression levels. Real-time PCR (RT-PCR) was performed to evaluate the level of transcribed mRNA for both proteins in metastasized lungs. As shown in Figure 1, at 30 days post injection, similar ratios of PD-L1 to Her-2/neu RNA were observed in control mice and in mice actively immunized with the multi-peptide vaccine. However, 45 days after tumor cell injection, the ratio of PD-L1 to Her-2/neu RNA was significantly higher in vaccinated mice than in control mice, indicating a significant upregulation of PD-L1 and/or downregulation of Her-2/neu at this time point. The significantly increased ratio of PD-L1 to Her-2/neu RNA, 45 but not 30 days after tumor cell injection, is in line with our previous observation showing a delayed immunological response to vaccination [9].

**Figure 2:**
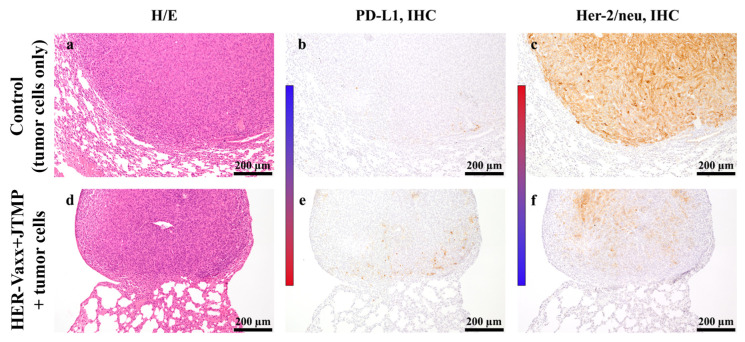
The protein expression status of PD-L1 and Her-2/neu in metastasized lungs of mice sacrificed at the 45-day post-injection time point. Representative images of consecutive lung sections of the mice sacrificed at the 45-day post-injection time point, after H/E staining (**a**,**d**), and single PD-L1 (**b**,**e**) and Her-2/neu (**c**,**f**) staining by IHC, are shown for the respective treatments; control (tumor cells only) and with the multi-peptide vaccine (HER-Vaxx + JTMP). The metastasis in the upper panel (untreated group), also located on the lung’s margin, compresses the lung tissue more than the smaller metastasis in the lower panel (treated group). The heat map bars emphasize the upregulated (blue to red) and downregulated (red to blue) expression of the stained proteins.

As mentioned above, in our previous study we showed that the vaccination of mice with HER-Vaxx resulted in significantly upregulated PD-L1 and downregulated Her-2/neu expression in lungs metastases [9]. In this study, consecutive sections of metastasized lungs of the control and vaccinated mice from the 45-day post-injection time point (Figure 1) were evaluated by IHC staining of PD-L1 and Her-2/neu, after H/E staining (Figure 2a,d), to examine whether the alterations in the protein expression were concomitant. The vaccination was shown to result in the upregulation of PD-L1 protein expression (Figure 2b,e) concomitant with the downregulation of Her-2/neu protein expression (Figure 2c,f) in the same tumors. The regions of the same tumor with increased levels of PD-L1 expression showed a loss of Her-2/neu expression.

**Figure 3:**
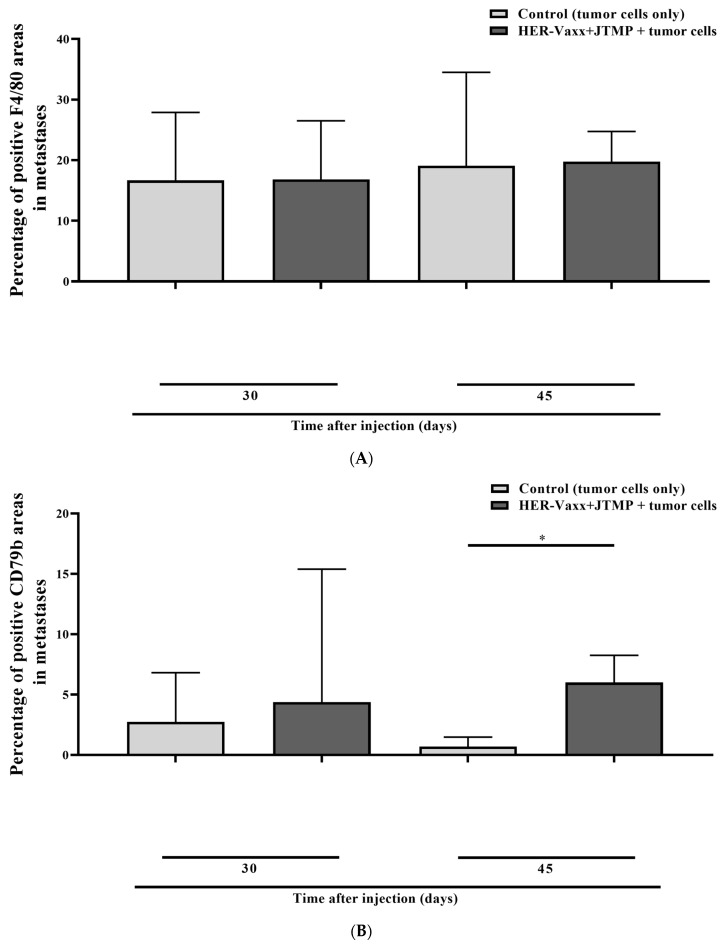
Percentage of positive F4/80 (macrophages) areas (**A**) and positive CD79b (B cells) areas (**B**) in the metastasized lungs of mice. Mice were immunized with a multi-peptide vaccine combining both HER-Vaxx and the mimotope of pertuzumab (JTMP), followed by tail-vein injection with tumor cells expressing human Her-2/neu, and sacrificed at 30 and 45 days following the tumor cell injection [8,9]. Based on F4/80 or CD79b single staining by IHC, the areas stained positive were assessed and are presented as percentages of tumor tissue in the graph’s bars. Up to 21 detected positive-stained areas were assessed and used for the statistical analyses. A representative IHC image, indicating the staining levels, is shown under each bar, scale bar = 60 µm. (*, *p* < 0.05).

For further evaluation, the metastasized lungs were stained for macrophages (F4/80) and B cells (CD79b). As shown in Figure 3A, no significant difference in the F4/80-positive areas was observed in the metastasized lungs of the control and vaccinated mice, at both time points, suggesting no increased infiltration of the lungs with macrophages at the evaluated time points. However, compared to the control mice, significantly more areas stained positive for B cells at the day-45 time point, but not on day 30, were observed in the vaccinated mouse lungs (Figure 3B), indicating the recruitment of B cells in the metastasized lungs following vaccination.

These results suggest an associated upregulation of PD-L1 expression in conjunction with the downregulation of Her-2/neu expression following Her-2/neu therapy with the examined multi-peptide vaccine in the employed preclinical setting. Furthermore, the association does not seem to be linked to infiltrating F4/80+ macrophages and CD79b+ B cells.

The above observation tempted us to evaluate the occurrence of the same phenomenon in the primary tumor of a patient enrolled in the Her-Vaxx phase Ib trial [10] who had disease progression at the end of the treatment and had developed a new Her-2/neu-negative metastatic lesion concomitant with the loss of Her-2/neu expression in the primary tumor [10].

### 2.2. Treatment with HER-Vaxx Is Associated with Upregulation of PD-L1 Expression in Concomitance with Downregulation of Her-2/meu Expression—Clinical Observation

The dose-escalation trial NCT02795988 for the evaluation of HER-Vaxx included an adult patient who was diagnosed with T4N3M1 stage IV Her-2/neu+++ adenocarcinoma of the stomach with four target lesions (2x liver and 2x lymph) and five non-target lesions and who received, in addition to cisplatin-5FU chemotherapy, a total of seven vaccinations (50 µg dose). During the course of the vaccination, the patient responded to the vaccine and generated high levels of vaccine-induced antibodies against Her-2/neu, with antibody levels rising from 30 ng/mL on day 0 to 2000 ng/mL after three vaccinations [10]. The strong antibody response in this patient, together with their cellular responses, reflected by high Th1 cytokine (IFNγ/IL-10, and TNFα/IL-10) ratios, correlated with the patient’s increase in progression-free survival and tumor reduction [10]. After 182 days of treatment, the patient had a partial response (PR) for target lesions with a 71% reduction in the sum of diameters (SOD) from 177 mm to 52 mm, and a complete response (CR) for four of the five non-target lesions. By day 266, the target lesion SOD (nadir) was reduced by 79% (from 177 to 37 mm). However, 350 days after treatment, the patient showed disease progression, and the primary tumor size increased by 16% to 43 mm, according to RECIST 1.1. The patient also developed a newly identified metastatic lesion in the stomach, which was Her-2/neu-negative [10], suggesting immune evasion of the metastatic tumor during Her-2/neu therapy. The patient’s primary tumor at the pre- and post-treatment time points was retrospectively assessed for PD-L1 and Her-2/neu expression.

**Figure 4:**
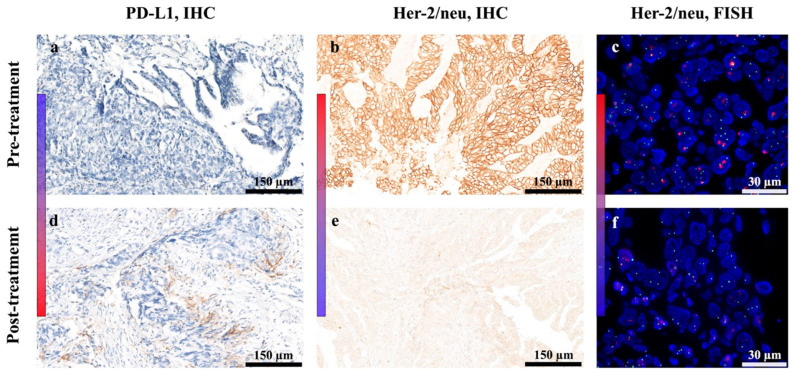
PD-L1 and Her-2/neu status in the primary tumor of the referenced patient. The levels of PD-L1 protein surface expression were assessed with IHC staining of the patient’s primary tumor before (**a**) and after treatment (**d**). The levels of Her-2/neu protein surface expression were assessed using immunohistochemistry (IHC) staining and by fluorescent in situ hybridization (FISH) of the patient’s formalin-fixed paraffin-embedded gastric primary tumor before (**b**,**c**) and after treatment (**e**,**f**). The heat map bars emphasize the upregulated (blue to red) and downregulated (red to blue) expression of the stained proteins.

The assessment of the pre-treatment (baseline) level of PD-L1 expression in the patient was negative (0%; CPS) (Figure 4a), whereas the Her-2/neu status was 3+ overexpression (IHC) (Figure 4b) with an Her-2/neu/CEP17 (FISH) ratio of 4.98 (Figure 4c). In contrast, post-treatment evaluation of PD-L1 and Her-2/neu expression in the patient’s primary tumor showed 5% PD-L1 expression (CPS) (Figure 4d) and an Her-2/neu status of 0 (IHC) (Figure 4e) with an Her-2/neu/CEP17 ratio of 1.63 (FISH; Figure 4f).

These results indicated the upregulation of PD-L1 and the downregulation of Her-2/neu in the reference patient, who had disease progression following Her-2/neu-targeting vaccination with HER-Vaxx.

## 3. Discussion

Trastuzumab has been shown to upregulate tumoral PD-L1 associated with the downregulation of Her-2/neu expression, leading to acquired resistance and immune evasion. In this study, we investigated the above association in both the preclinical and clinical settings after vaccination with the B-cell peptide-based vaccine HER-Vaxx targeting trastuzumab’s binding site. In both settings, a significant reduction in Her-2/neu in association with the upregulation of PD-L1 expression was observed.

The upregulation of PD-L1 following treatment with trastuzumab and recruitment of immune effector cells, mediating antibody-dependent cell cytotoxicity (ADCC), and the stimulation of IFNγ secretion [15] as a potential mechanism of resistance to the mAb [15], have been reported in gastric malignancies [16]. In parallel, the loss of Her-2/neu expression following treatment with trastuzumab has been reported among breast cancer patients [17] and patients with gastric or gastroesophageal cancers [18], resulting in the escape of tumor cells from treatment and the survival advantage of Her-2/neu-negative cancer cells. Here, we demonstrated that vaccination-based Her-2/neu-targeting therapy results in the upregulation of PD-L1 and the downregulation of Her-2/neu expression in both preclinical and clinical settings. It has been reported that overexpression of Her-2/neu is associated with the downregulation of MHC-I molecules in tumor cells [19], with the latter being essential for tumor recognition by cytotoxic T-lymphocytes (CTLs). The role of IFNγ, produced by CTLs, and the induction of PD-L1 overexpression have also been demonstrated [20]. Currently, it is unknown whether the upregulation of PD-L1 in the patient’s tumor referenced in this study is associated with the upregulation of MHC-I; however, we demonstrated increased levels of Her-2/neu-specific IFNγ production in the patient in a phase Ib trial [10]. Thus, we speculate that the increased levels of IFNγ in the tumor microenvironment may have led to the upregulation of PD-L1 in association with the loss of Her-2/neu expression in the patient’s tumor.

In recent studies, the effect of Her-2/neu targeting by trastuzumab, the inhibition of the Akt signaling pathway, and the consequent upregulation of PD-L1 expression have been speculated [15]. In our phase Ib trial, we reported that vaccination with HER-Vaxx resulted in the induction of polyclonal Her-2/neu-specific antibodies in patients [10]. The induced antibodies, including those in the patient referenced in this study, could inhibit intracellular Her-2/neu phosphorylation and the downstream phosphorylation of the Akt (Thr 308) signaling pathway in gastric cancer cells (NCI-N87) [10]. Thus, these observations suggest that the upregulation of PD-L1, shown here in preclinical and clinical evaluations, is the direct result of Her-2/neu-targeting therapy in combination with the effects of the tumor microenvironment. The preclinical setting in our study was based on the evaluation of Her-2/neu lung metastases that originated from the mammary carcinoma cells D2F2/E2 transfected for the expression of Her-2/neu (transgene) [21]. The loss of Her-2/neu and the upregulation of PD-L1 expression were observed in the clinical setting, and the relative increase in tumors expressing PD-L1 compared to those expressing Her-2/neu, as well as the relative increase in the respective RNA in the mouse model, point in the same direction. Along this line, in a recent study, the direct role of the Akt signaling pathway and the regulation of PD-L1 expression were shown [22]. Furthermore, the same study showed the expression of PD-L1 at both the transcriptional (mRNA) and translational (protein) levels in epithelial and endothelial cell lines [22], which supports our results showing concomitantly increased mRNA transcripts and surface-expressed PD-L1 (Figure 1 and Figure 2). 

Pertuzumab, which binds to the dimerization loop in extracellular domain II of Her-2/neu, has shown a synergistic effect in combination with trastuzumab on the clinical outcome of patients with Her-2/neu-overexpressing breast cancers [23]. Since both trastuzumab and pertuzumab inhibit intracellular Her-2/neu phosphorylation, as well as the phosphorylation of the downstream Akt signaling pathway [24], the upregulation of PD-L1 that was observed following treatment with our multi-peptide vaccine might be the result of a broader binding spectrum of the induced antibodies, thus leading to stronger therapeutic pressure on the receptors expressed on tumor cells [9].

The upregulation of PD-L1 expression upon cellular activation has been reported in lymphocytes, dendritic cells, and myeloid cells, including macrophages [25]. In our study, IHC indicated moderate to strong expression of PD-L1 in the metastases of the vaccinated animals. Staining of the metastatic tumor in the animals for Her-2/neu and PD-L1 showed increased expression of PD-L1 and the loss of Her-2/neu expression in the same region of the tumor cells (Figure 2e,f), suggesting a direct link between the proteins’ altered expression in the tumor cells. This is further supported based on the staining of the metastasized mouse lungs showing no increased levels of macrophages.

Based on the RT-PCR evaluation, the ratio of PD-L1 to Her-2/neu RNA was decreased in the control, i.e., non-immunized, mice. Two factors may explain this decrease: (1) in this group, the mice were not treated with the multi-peptide vaccine, i.e., Her-2/neu was not targeted, and therefore the tumor cells may have continued expressing the receptor, which is in direct correlation with increased RNA levels; (2) as mentioned above, it has been shown that Her-2/neu-targeting therapy with trastuzumab increases the PD-L1 expression. The lack of targeting the receptor in the control mice presumably kept the expression of PD-L1 and its RNA transcript unchanged, or even somewhat reduced, as the Her-2/neu expression leaves the mitotic activity unchanged [26]. These factors together may reflect the reduced ratio of PD-L1 to Her-2/neu RNA in the control mice from the day-30 to day-45 time points. Additionally, no increase in the ratio from day 30 to day 45 in the HER-Vaxx+JTMP group was observed, and we speculate that it might have been due to the interval between the two examined time points when the ratio was assessed. An assessment at a later time point, e.g., one or two weeks after the day-45 time point, could result in a further increased ratio. Support for this speculation is our earlier findings [9], which showed an enhancement of the vaccination effect over time. However, all in all, the results of this study indicate a significant difference in the evaluated ratio between the two groups at the examined day-45 time point, suggesting a direct role of the vaccination.

The role of infiltrating vaccine-induced B cells and T cells in tertiary lymphoid structures and better prognosis has been demonstrated repeatedly [27,28]. In addition to the important role of B cells in humoral immunity, the prognostic role of B-cell infiltration of tumors in different cancers has been shown [29,30]. HER-Vaxx is based on B-cell epitope peptides activating naïve B cells which mature toward the production of Her-2/neu-specific antibodies [10,31]. The formulated vaccine also includes the carrier protein CRM197, comprising T cell epitopes, which activate T cells and subsequently enhance the production of peptide-specific antibodies by CD4 T helper cells. Based on our previous preclinical and clinical observations, vaccination with HER-Vaxx induces production of IFNγ [31,32]. In the current study, the staining of the mouse lungs for B cells indicated a significant increase in vaccine-induced infiltration of B cells. While we speculate that the detected B cells are vaccine-induced with the capacity to reduce the tumors’ size, further evaluations are ongoing to examine whether the detected B cells are PD-L1-positive, i.e., regulatory B cells, which have been reported in various cancers [33,34,35].

The effectiveness of HER-Vaxx was recently demonstrated in first-line patients with Her-2/neu-positive advanced GC, showing tumor regression and overall survival benefit [11,12,13]. GC is a highly heterogeneous malignancy, with Her-2/neu overexpression ranging from 26% to 79% [36]. Thus, a more adapted strategy for combination therapy based on the level of Her-2/neu expression might be required. An adapted strategy might also be required in cases of tumors that already have a low expression level of PD-L1 at baseline. In addition to the intra-tumoral heterogeneity, the effect of intervals between Her-2/neu-targeted therapies and Her-2/neu expression has been shown and is of importance [17], which, based on the results in our study, can in turn affect tumoral PD-L1 expression. Therefore, a combination therapy to target Her-2/neu and PD-L1 based on a sequential schedule might be considered. Our study suffers from two limitations: (1) The observation from the reported clinical setting is based on the primary tumor of one patient only. Therefore, evaluation of additional cases for the expression of Her-2/neu and PD-L1 is necessary, and planned, to further support the proposed combination immunotherapy by vaccination against both targets; (2) The upregulation of other ICs, such as T-cell Immunoglobulin and Mucin containing protein-3 (TIM-3), Lymphocyte activation gene-3 (LAG-3), and B and T Lymphocyte Attenuator (BTLA), was not evaluated in this study. The upregulation or co-expression of these ICs, expressed on intra-tumoral CD8 T-cells, results in T-cell exhaustion, reduced avidity and, consequently, tumor cells’ immune evasion [37,38,39,40]. The combination of IC inhibitors and targeted drugs has shown significant synergistic effects [41,42,43], and several clinical trials are ongoing to evaluate the combination of Her-2/neu-targeted therapy and ICIs in breast cancers [44]. However, firstly, since obviously the occurrence of vaccinated patients who progress and develop new metastatic lesions with the loss of Her-2/neu and upregulation of PD-L1, due to the reported type of immune evasion, are relatively rare, we have to wait for such cases to occur. Secondly, similar observations based on the preclinical setting further emphasize the potential role of Her-2/neu-targeted vaccination in promoting tumoral upregulation of PD-L1 and downregulation of Her-2/neu expression and stress a targeted combination therapy.

## 4. Materials and Methods

### 4.1. Cell Line and Culture Conditions

D2F2/E2 cells, a BALB/c mouse cell line derived from a mammary carcinoma line that was transfected with the human Her-2/neu gene (transgene) for stable expression of the receptor [21], were kindly provided by Prof. Wei-Zen Wei (Karmanos Cancer Institute, Wayne State University School of Medicine, Detroit, MI, USA). The cells were maintained in high-glucose DMEM (supplemented with FBS (10%), NCTC 109 (10%) medium, L-glutamine (200 mM), PenStrep (100 units/mL penicillin, 100 μg/mL streptomycin), Sodium Bicarbonate (5%), 2-mercaptoethanol (10 mM), and Sodium bicarbonate (2%).

### 4.2. Preclinical Setting

#### 4.2.1. Animals and Immunization Settings

Female BALB/c mice aged 6–8 weeks at the time of delivery were purchased from Charles River (Germany), maintained under conventional housing conditions, and used in the experiments detailed below. The experiments were approved by the Animal Experimentation Committee of the Medical University of Vienna and the University of Veterinary Medicine and by the Austrian Federal Ministry of Science and Research (BMWF-66.009/0136-WF/V/3b/2017).

Experimental setting: Two groups of mice (*n* = 8/group) were subcutaneously injected (actively immunized) with the multi-peptide vaccine HER-Vaxx and the mimotope of pertuzumab JTMP (conjugated to CRM197 and administered Montanide ISA-51-VG (Seppic, Courbevoie, France). The multi-peptide vaccine was prepared as described previously [9]. One week after the third subcutaneous injection/immunization, 5 × 10^4^ of the tumor cells D2F2/E2 were injected into the tail vein of the mice. Control setting: Two additional groups of mice (*n* = 8/group) received PBS by subcutaneous injection and then injected in the tail-vein with 5 × 10^4^ D2F2/E2 cells. One group of mice from each setting was sacrificed at a 30-day post-injection time point, and similarly at a 45-day post-injection time point. Mouse lungs were excised and evaluated for PD-L1 and Her-2/neu expression using Real-Time Polymerase Chain Reaction (RT-PCR) (days 30 and 45) and IHC staining (day 45).

#### 4.2.2. Pathohistological Assessment

For histological examination, the right lung lobes were excised, fixed in a 4% buffered formaldehyde solution, and embedded in paraffin wax. Sections of 2–3 µm thickness, covering the entire lung, were cut on a sliding microtome and stained with H/E. IHC staining was performed using primary antibodies against Her-2/neu, PD-L1, F4-80, and CD79b using an autostainer (Lab Vision AS 360, Thermo Scientific, Fremont, CA, USA). Details regarding primary antibodies, dilution, antigen retrieval, secondary antibodies, chromogen, and counterstain are presented in Supplementary Table S1.

An experienced pathologist blinded to the treatment regimens descriptively evaluated the HE-stained lungs using an Olympus BX-53 microscope (Olympus, Tokyo, Japan). Images of Her-2/neu- and PD-L1-stained lung sections were taken with an Olympus DP26 camera (10× objective lens) and evaluated using the open-source software FIJI in ImageJ, version 1.54h (RRID: SCR_003070) [45]. Adobe Photoshop (RRID: SCR_014199) 2021 was used for white balance and arrangement of representative images. To quantify CD79b and F4/80 expression, slides were scanned using a PANNORAMIC Scan II from 3DHISTECH (Budapest, Hungary) and saved as MRXS files. Analysis of IHC staining was performed with QuPath (version 0.4.3) by a pathologist blinded to the treatment regimens. For each lung, all metastases were annotated and the area above a defined DAB threshold was measured. Representative pictures for figures were exported using the snapshot function of CaseViewer (Build, version 2.4.0.119028).

#### 4.2.3. Assessment of PD-L1 and Her-2/neu Expression using RT-PCR

Portions of the lungs from the control and immunized mice were homogenized and used for the isolation of total RNA using an RNeasy kit (QIAGEN, Hilden, Germany) according to the manufacturer’s instructions. Isolated RNA was quantified using an ND-1000 Spectrophotometer (Nanodrop Technologies Inc., Wilmington, NC, USA). One microgram of total RNA was used for the preparation of cDNA using an iScript™ cDNA Synthesis Kit (Bio-Rad, Hercules, CA, USA) as specified by the manufacturer.

RT-PCR was performed to measure the relative mRNA expression of PD-L1 and Her-2/neu. The mouse housekeeping gene β-actin was used as an internal control to measure the relative expression of the examined genes using the 2^−ΔCt^ method. Briefly, SsoAdvanced™ Universal SYBR Green Supermix kit (Bio-Rad, USA) was used for the RT-PCR reactions, using the following primers: mouse PD-L1 (Accession #NM_021893.1; Forward: 5′-TGCGGACTACAAGCGAATCACG-3′, Reverse: 5′-CTCAGCTTCTGGATAACCCTCG-3′), human Her-2/neu (Accession #NM_001382796.1; Forward:5′-GCACAGACATGAAGCTGCG-3′, Reverse: 5′-GTGGGCAGGTAGGTGAGTTC-3′), and mouse β-actin (Accession #XM_021163894.2; Forward: 5′-GATCAAGATCATTGCTCCTCCTGA-3′, Reverse:5′-CAGCTCAGTAACAGTCCGCC-3′). PCR reactions, in total volumes of 10 µL, included 5 µL SsoAdvanced SYBR Green reaction mix (Bio-Rad, Hercules, CA, USA), 0.25 µL of each primer at a concentration of 20 µM, 3 µL of nuclease-free water, and 1.5 µL of the prepared cDNA. RT-PCR reactions were carried out on a CFX Connect Real-Time System (Bio-Rad) under the following conditions: 1 cycle at 95 °C for 2 min, 40 cycles at 95 °C for 15 s and 60 °C for 30 s. This was followed by a dissociation stage (melting curve) at 65 °C for 30 s and cycles of 5 s starting at 65 °C and increasing by 0.5 °C per cycle. Following amplification, Ct values were obtained using CFX Manager software 3.1 (Bio-Rad).

### 4.3. Clinical Setting

#### 4.3.1. The Phase Ib Dose-Escalation Trial (IMU.ACS.001, NCT02795988)

The completed trial, NCT02795988, evaluated different doses of HER-Vaxx (10 µg, 30 µg, and 50 µg of the vaccine peptide P467) and enrolled 14 patients with Her-2/neu-overexpressing metastatic or advanced adenocarcinoma of the stomach or GEJ. Among the enrolled patients, 11 (10 µg, *n* = 3; 30 µg, *n* = 5; 50 µg, *n* = 3) were evaluated for the assessment of tumor responses [10]. The trial was a multicenter study conducted in Georgia, Moldova, Taiwan, and Thailand [10]. Pre- and post-treatment biopsies from the primary tumor sites of the patient referenced in this study were assessed for PD-L1 and Her-2/neu expression using immunohistochemistry (IHC), fluorescence in situ hybridization (FISH), and combined positive score (CPS).

#### 4.3.2. Biopsies from the Vaccinated Patient

In a clinical study, one patient showed disease progression. Four-micrometer-thick slides were cut from the formalin-fixed paraffin-embedded (FFPE) blocks with the patient’s biopsies before treatment on day 0 and at the end of the treatment on day 350 (following three single-dose vaccine injections and four booster vaccinations for long-term maintenance). PD-L1 immunohistochemical (IHC) staining was carried out using a Dako PD-L1 immunohistochemistry 22C3 pharmDx assay (Dako, Carpinteria, CA, USA), including appropriate controls, according to the manufacturer’s instructions. PD-L1 assessment was based on CPS, which had been similarly applied in two recent phase III trials evaluating the effect of nivolumab [46] and pembrolizumab [47] in patients with advanced GC and esophageal adenocarcinoma, respectively. IHC staining for Her-2/neu was performed using a 4B5 mAb (Ventana, Tucson, AZ, USA) for the BenchMarkULTRA system (Ventana). Images of Her-2/neu- and PD-L1-stained lung sections were obtained using a Philips IntelliSite Pathology Solution with an Ultra-Fast Scanner. Fluorescence in situ hybridization (FISH) for the evaluation of Her-2/neu gene amplification was performed as described previously [10].

### 4.4. Statistical Analysis

In the animal experiment, subgroups of control animals and vaccinated animals, both injected with 5 × 10^4^ tumor cells, were sacrificed on days 30 and 45 after injection. RNA levels of PD-L1 and Her-2/neu were measured relative to β-actin expression. The ratio of the relative RNA expression of PD-L1 to that of Her-2/neu was calculated. These values were statistically evaluated using a generalized linear model with group (controls/vaccinated) and days (30 and 45) as between factors, together with their interaction, assuming a log-normal distribution. Comparisons of the ratios of PD-L1 to Her-2/neu between the groups at the two time points were obtained from linear contrasts by applying Sidak correction. Similarly, the ratio of PD-L1- to Her-2/neu-positive tumors in the lungs was statistically compared to that of control animals by applying a generalized linear model and linear contrasts. Statistical significance was set at *p* < 0.05. The analysis was performed using Stata 17 (StataCorp, College Station, Tulsa, OK, USA).

## 5. Conclusions

Overall, the observed tumoral upregulation of PD-L1 and downregulation of Her-2/neu expression, based on the comparison of the same vaccine in both preclinical and clinical settings, is clinically relevant for the elimination of tumor cells with a PD-L1-positive/Her-2/neu-negative status, preventing new metastasis and immune evasion. As mentioned above, the role of other ICs on T cell response and their inclusion in combination therapy is also indisputable. In line with this, we have shown that targeting PD-1 in combination with HER-Vaxx enhances the vaccine’s anti-tumor effect in mice with Her-2/neu-expressing solid tumors [48]. Based on these results and the observation shown in the current study, two clinical trials have been planned (Imugene Limited, Sydney, Australia) to assess the following: (1) HER-Vaxx in combination with chemotherapy or the anti-PD-1 antibody pembrolizumab in patients with Her-2/neu-overexpressing gastric cancer who have failed treatment with trastuzumab, and (2) HER-Vaxx in combination with chemotherapy +/− the anti-PD-L1 antibody avelumab in patients with Her-2/neu-overexpressing gastric cancer.

## Data Availability

The data presented in this study are available upon request from the corresponding authors.

## Supplementary Materials

The following supporting information can be downloaded at: https://www.mdpi.com/article/10.3390/ijms25010287/s1.

## Abbreviations

ADCC	Antibody-dependent cell cytotoxicity
CD4	Cluster of differentiation 4
CD79b	Cluster of differentiation 79b
CPS	Combined üositive score
CR	Complete response
CTL	Cytotoxic T-lymphocytes
FISH	Fluorescence in situ hybridization
IC	Immune checkpoint
IHC	Immunohistochemistry
IFNγ	Interferon gamma
MHC-I	Major histocompatibility complex
mRNA	Messenger RNA
PBS	Phosphate-buffered saline
PD-1	Programmed cell death 1
PD-L1	Programmed cell death ligand 1
RNA	Ribonucleic acid
RT-PCR	Reverse transcription polymerase chain reaction
SOD	Sum of diameters
