# Supplementary material for: Preclinical and Clinical Observations Implying Combination Therapy to Enhance the Efficacy of the Her-2/neu B-Cell Peptide-Based Vaccine HER-Vaxx and to Prevent Immune Evasion
Source: Int J Mol Sci. 2023 Dec 24;25(1):287. doi: 10.3390/ijms25010287 (PMC10778754; doi:10.3390/ijms25010287)
Supplement: Supplementary file 1 [file ijms-25-00287-s001.zip › JTobias_Supplementary Materials; 2023-11-20.pdf]

Supplementary Materials

Supplementary Table

Table S1- Details regarding primary antibodies, dilution, antigen retrieval, secondary antibodies, chromogen, and counterstain, used for the pathohistological assessments.

Antibody	Dilution	Antigen retrieval	Detection System	Immunostaining reagent	Counterstain
**PDL1**<br>**CST#64988**	1: 200	Heating in<br>citrate buffer<br>pH 6	secondary antibody conjugated to enzyme labelled polymer<br>(Bright Vision Rabbit HRP KL DPVR 110 HRP)	DAB (DAB Quanto #_TA-125-QHDX)	hematoxylin
**Her2**<br>**CST#2242**	1: 100	Heating in<br>Tris-EDTA buffer<br>pH 9	secondary antibody conjugated to enzyme labelled polymer<br>(Bright Vision Rabbit HRP KL DPVR 110 HRP)	DAB (DAB Quanto #_TA-125-QHDX)	hematoxylin
**F4/80**<br>**CST#70076**	1: 1000	Heating in<br>citrate buffer<br>pH 6	Strept-Avidin biotin complex method with horse radish peroxidase<br>(Abcam ab64269)	DB (DAB Quanto # TA-125-QHDX)	hematoxylin
**CD79b**<br>**CST#96024**	1: 250	Heating in<br>Tris-EDTA buffer<br>pH 9	secondary antibody conjugated to enzyme labelled polymer<br>(Bright Vision Rabbit HRP KL DPVR 110 HRP)	DAB (DAB Quanto #_TA-125-QHDX)	hematoxylin
